# Inline Measurement of Particle Concentrations in Multicomponent Suspensions using Ultrasonic Sensor and Least Squares Support Vector Machines

**DOI:** 10.3390/s150924109

**Published:** 2015-09-18

**Authors:** Xiaobin Zhan, Shulan Jiang, Yili Yang, Jian Liang, Tielin Shi, Xiwen Li

**Affiliations:** 1 State Key Laboratory of Digital Manufacturing Equipment and Technology, Huazhong University of Science and Technology, Wuhan 430074, China; E-Mails: hustzhan@163.com (X.Z.); yangyili5727@163.com (Y.Y.); liangjian5282@126.com (J.L.); tlshi@hust.edu.cn (T.S.); 2 Tribology Research Institute, National Traction Power Laboratory, Southwest Jiaotong University, Chengdu 610031, China; E-Mail: voyagejsl@sina.com

**Keywords:** ultrasonic sensor, particle concentration, multicomponent suspensions, inline measurement, LS-SVM

## Abstract

This paper proposes an ultrasonic measurement system based on least squares support vector machines (LS-SVM) for inline measurement of particle concentrations in multicomponent suspensions. Firstly, the ultrasonic signals are analyzed and processed, and the optimal feature subset that contributes to the best model performance is selected based on the importance of features. Secondly, the LS-SVM model is tuned, trained and tested with different feature subsets to obtain the optimal model. In addition, a comparison is made between the partial least square (PLS) model and the LS-SVM model. Finally, the optimal LS-SVM model with the optimal feature subset is applied to inline measurement of particle concentrations in the mixing process. The results show that the proposed method is reliable and accurate for inline measuring the particle concentrations in multicomponent suspensions and the measurement accuracy is sufficiently high for industrial application. Furthermore, the proposed method is applicable to the modeling of the nonlinear system dynamically and provides a feasible way to monitor industrial processes.

## 1. Introduction

Inline measurement of the component concentrations is an important approach to supervise industrial processes. Based on the concentration information, the process controls can be implemented and the stable product quality, the processing efficiency and the energy saving can be ensured [1]. Nevertheless, the measurement of component concentrations in multicomponent mixtures cannot be easily achieved through a low-cost and inline technique. So far, several methods have been proposed to measure component concentrations. The sampling method [2] is used to measure the local solid concentration based on separate solid and liquid phases, so it is not suitable for inline measurement. The multi-physics field combined methods [3,4] are accurate, but they confront one with the problems of high cost and complexity. It is still necessary to develop simple and low-cost methods. Ultrasonic sensor can satisfy the requirements of good robustness, high accuracy, inline measurement, safety and low maintenance in industrial processes [5]. In recent years, the ultrasonic sensor has been successfully used to measure the concentration of mixtures. For binary mixtures, the ultrasonic features (e.g., ultrasonic attenuation and velocity) are directly correlated with concentrations [6,7]. However, it is very difficult or even impossible to derive a physical model to characterize the relationship between ultrasonic features and component concentrations in multicomponent mixtures [8].

The variations of component concentrations have effects on the physical properties of multicomponent mixtures, including density, viscosity and bulk modulus [9]. So the ultrasonic waves (amplitude and shape) transmitted through the multicomponent mixtures will also change along with the component concentrations, and, in turn, it is possible to measure the component concentrations through analyzing the ultrasonic signals. Some researchers have studied the possibility that the ultrasonic sensor is used to measure the component concentrations in multicomponent solutions. Resa *et al.* [10] have proposed the monitoring of alcoholic fermentation processes based on the combination of ultrasound velocity and semi-empirical models. Krause *et al.* [1,11] have put forward a multivariate regression method for simultaneous detection of sugar and ethanol concentrations in aqueous solutions. Schafer *et al.* [8] reported that the partial least square (PLS) model could be used to estimate the concentration of sodium chloride in an aqueous solution by analyzing spectra of ultrasonic pulses. This research suggests that it is feasible to measure component concentrations in some solutions using ultrasonic sensors by means of multivariate analysis, but the ultrasonic measurement of component concentrations in multicomponent suspensions has been rarely reported.

The ultrasonic transmission mechanism of multicomponent suspensions is far more complex than that of multicomponent solutions [12]. In multicomponent suspensions, the ultrasonic features and the component concentrations exhibit a nonlinear relationship because of the strong interactions among components. The statistical multivariate methods, such as the PLS model and the multiple linear regression (MLR) model, are not necessarily good options for modeling the nonlinear relationship between the features and target values [13]. While the advanced models (such as the artificial neural network (ANN) approach and the LS-SVM model) are commonly applied to nonlinear systems as alternative solutions. Li *et al.* [14] presented an ultrasonic method to measure the liquid pressure and in that work, the ANN was employed to improve the measurement accuracy. The ANN has a disadvantage that the available sample set should be considerably large for the purpose of effective ANN training, while the LS-SVM model can build the model using a small sample set [15]. In addition, with the help of kernels, the LS-SVM model is capable of modeling the nonlinear system in high-dimensional feature spaces with fewer training data [16]. The LS-SVM has been effectively applied to inline estimate the biomass in fermentation process [17], quantify the common adulterants in powdered milk [18], and predict the milk-to-plasma drug concentration ratio [19], *etc*. Although the establishment of a LS-SVM model is time-consuming, once the model is established, the analysis can be completed within a short time [20]. Therefore, the LS-SVM model is a promising technique for inline measurement of the particle concentrations in multicomponent suspensions.

Ultrasonic signals contain a wealth of information, but some information doesn’t contribute to the prediction of particle concentrations. Therefore, it is necessary to exclude non-informative and unimportant features that strongly affect the model stability [11]. Then, the robustness and accuracy of model can be improved and while the complexity of model can be reduced [21].

This paper is aimed to develop a new inline measurement system for simultaneous measurement of two particle concentrations in ternary suspensions based on ultrasonic sensor and the LS-SVM model. The features are collected from ultrasonic signals of time and frequency domains at first. Then the LS-SVM model is tuned, trained and tested to realize rapid inline prediction of the particle concentrations through some ultrasonic features. In addition, the comparative analysis between the PLS model and the LS-SVM model is conducted to give some insight into the two methods. Finally, the optimal LS-SVM model and the optimal feature subset are applied to inline measurement of particle concentrations in the mixing process. The proposed method is simple and easy to implement and can be extended to detecting the mixtures of more components. As far as the authors are concerned, this is a pioneering project in the field of concentration measurements of multicomponent suspensions, which makes it possible to carry out inline monitoring for production processes and product quality in multicomponent suspensions with only one ultrasonic sensor.

## 2. Materials and Methods

### 2.1. Measurement Apparatus

**Figure 1:**
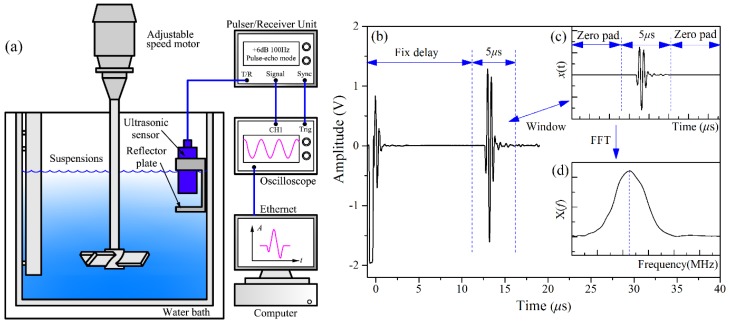
(**a**) Schematic diagram of the ultrasonic system for the measurement of suspensions concentration; (**b**) The full time domain signals of pure water; (**c**) The windowed, averaged and zero-padded time domain signals of (**b**); (**d**) The frequency spectrum of (**c**).

The experimental setup is plotted schematically in Figure 1a. The suspensions are contained in a transparent flat-bottomed cylindrical tank, with the inner diameter of 120 mm. The tank is immersed in a circulating thermostatic water bath to keep the constant temperature of suspensions at 30 °C, with accuracy of 0.05 °C. To ensure the homogeneity of suspensions, the suspensions are constantly stirred with a four-bladed pitched blade turbine (PBT-4) impeller. The ultrasonic system is composed of an ultrasonic broadband sensor (with the center frequency of 2.25 MHz) and an ultrasonic pulser/receiver unit in the pulse-echo mode. The ultrasonic sensor is parallel to the reflector plate, and their distance (*l*) is 9.9 mm. The ultrasonic pulse travelled through the suspensions is reflected from the reflector plate and returns back to the sensor. The arrangement of sensor to be suspended in slurry is intrusive in nature, but the signals of maximum strength can be received in this way [22].

The signals are acquired with an interval of 1s using a Tektronix (Beaverton, OR, USA) DPO7054 oscilloscope and processed in a computer via Ethernet. The oscilloscope is triggered at the same point of the synchronizing signals for each acquisition. The first reflected wave in the time domain is windowed over 5 μs, and 300 windowed signals are averaged. The resulting signal is denoted as *x*(*n*), where *n* = 1, 2, …, N and N stands for the number of sampling points. Afterwards, the signal *x*(*n*) is zero-padded and mapped to the frequency domain using the fast Fourier transform (FFT) algorithm. The resulting frequency domain signal is denoted as *X*(*k*), where *k* = 1, 2, …, M and M represents the number of zero-padded sampling points. The signal processing steps of pure water are shown in Figure 1b and c. As can be seen in Figure 1d, the center frequency is approximately 2.25 MHz, and the signal-to-noise ratio (SNR) is sufficiently high in the range of 0–6 MHz. Hence, the frequency spectrum *X*(*k*) in the bandwidth of 0–6 MHz is used to extract the frequency domain features.

### 2.2. Analysis Method

**Figure 2:**
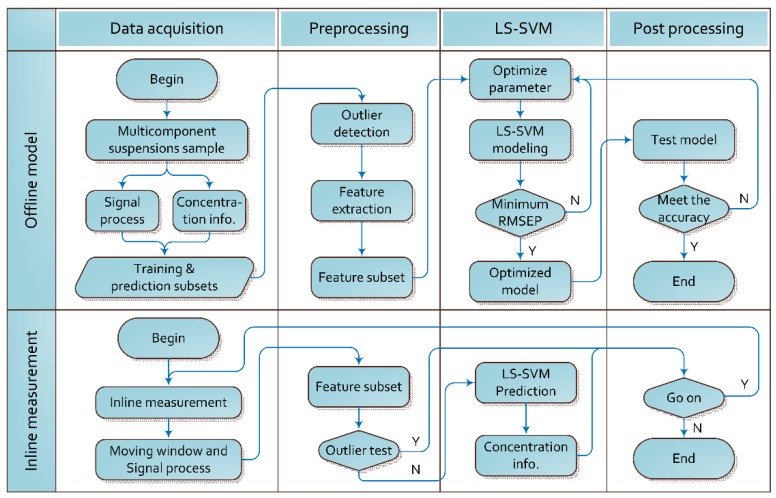
A flow chart of the whole process of modeling and inline measurement.

A two-stage architecture is proposed to improve the prediction accuracy and generalization performance of concentration measurement. In the first stage, the ultrasonic signals are analyzed and processed. The optimal feature subset that contributes to the best model performance is selected based on the importance of features, and the LS-SVM model is tuned, trained and tested with different feature subsets to obtain the optimal model. In the second stage, the optimal LS-SVM model with the optimal feature subset is applied to inline measure the particle concentrations in the mixing process. All necessary steps are summarized in Figure 2 and briefly introduced as follows:

1. Signal pre-processing. Ultrasonic signals are windowed, averaged, zero-padded and mapped to the frequency domain to obtain the time and frequency domain features. Due to the relatively large differences, the features have to be auto-scaled by zero-mean normalization method before further analysis.
2. Sample outliers. To reduce the signal noises and enhance the robustness of model, the outlier detection is very important. In inline applications, the outlier detection can determine whether the measurement is within the applicable scope of the model or not.
3. Feature extraction. The feature extraction is completed by analyzing the importance of features in the model. A commonly-used method to calculate the importance of features is a combination of the scaled regression coefficients (SRC) and variable importance in the projection (VIP) [11,23], in which SRC is calculated automatically whilst constructing the regression model and VIP is based on calculating the projection part of single variable in relation to the target values.
4. Model building and testing. The LS-SVM parameters are optimized at first, and then the model is trained and tested using the training and prediction subsets, respectively. The most appropriate model that produces the smallest prediction error can be obtained.
5. Inline measurement. The optimized model with the optimal feature subset is employed for inline measurement of particle concentrations of intentionally-designed samples.

The LS-SVM model is the core of the analysis procedure. The details of LS-SVM algorithm can be found in the literatures [16]. The final LS-SVM model can be expressed as follows:

(1) $$y\left(x\right)=\sum_{i=1}^{N}\alpha_{i}K\left(x,x_{i}\right)+b$$

where *α_i_* is Lagrange multiplier, *b* is the bias value, *K*(*x*, *x_i_*) is the kernel function and *x_i_* is the input data.

The optimal feature subset, the proper kernel function and the best kernel parameters are three key factors of the LS-SVM model. In this study, the selected ultrasonic features are employed as the input data of the LS-SVM model to reduce the training time and improve the prediction accuracy. RBF kernel is used as the kernel function of the LS-SVM model, because it is a nonlinear function that gives a good prediction performance under general smoothness assumptions. The RBF kernel can be expressed as:

(2) $$K\left(x,x_{i}\right)=\exp\left(\frac{-{||x-x_{i}||}^{2}}{\sigma^{2}}\right)$$

where *σ* is the RBF kernel parameter.

The regularization parameter λ and the width parameter σ^2^, play an important role in building a LS-SVM model with the RBF kernel featuring high prediction accuracy and stability [24]. In this study, an iterative grid search technique with 10-fold cross validation is employed to find out the optimal combination of (λ, σ^2^). After the three key factors are determined, the LS-SVM model is developed and the prediction performance is evaluated with training and prediction subsets, respectively.

The primary indicators for the model performance are the root mean square error (RMSE) and the coefficient of determination (*R*^2^). RMSE reflects the residual errors and provides a global idea of the difference between the observed and predicted values, which is expressed in the same unit as the target values [25]. In the paper, the RMSE of cross-validation (RMSEV) is used to decide the model parameters. However, it may lead to the over-fitting of the final model structure, so the RMSE of prediction subset (RMSEP) is used to check the final model. *R*^2^ reflects the goodness of fit of the model.$\;R_{p}^{2}$ stands for the *R*^2^ of prediction subset. They are given by:

(3) $$\text{RMSE}=\sqrt{\frac{1}{m}\sum_{i=1}^{m}{\left({\hat{Y}}_{i}-Y_{i}\right)}^{2}}$$

(4) $$R^{2}=1-\frac{\sum_{\text{i}=1}^{m}\left(Y_{i}-\hat{Y}\right)}{\sum_{\text{i}=1}^{m}\left(Y_{i}-\bar{Y}\right)}$$

where $Y_{i}$ and ${\hat{Y}}_{i}$ are the known target value and the corresponding predicted value in the sample *i* respectively, $\bar{Y}=\frac{1}{m}\overset{m}{\underset{i=1}{\sum }}Y_{i}$ is the mean of the known target, *m* is the total number of samples.

### 2.3. Sample Preparation

**Figure 3:**
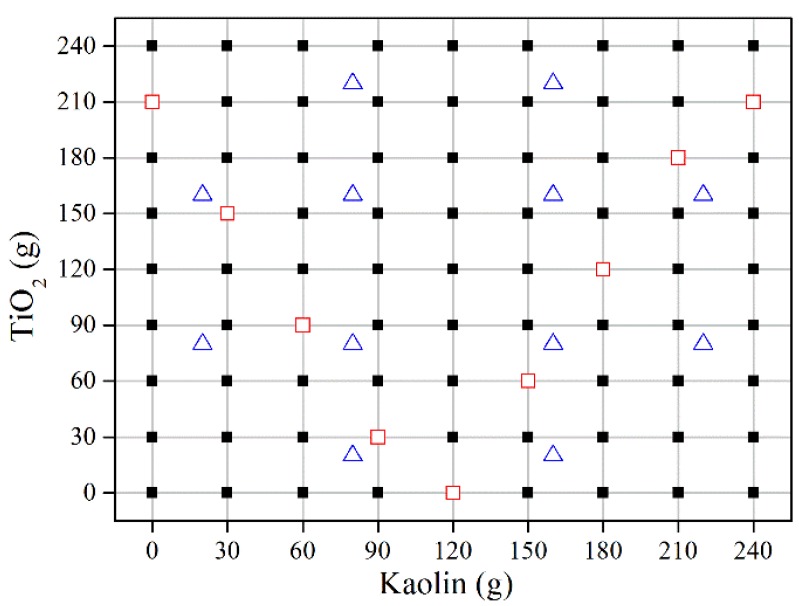
The experimental design of the samples: the black solid squares represent the training subset; the red hollow squares stand for the prediction subset; the blue triangles denote the inline test subset.

The multicomponent suspensions are composed of pure water, titanium dioxide (TiO_2_, with an average particle size of 19 μm and a density of 4260 kg/m^3^) and Kaolin (with an average particle size of 12 μm and a density of 2930 kg/m^3^). Both TiO_2_ and Kaolin are prepared in nine groups from 0 g to 240 g, with an interval of 30 g. Seven hundred grams of pure water is added into possible combinations of TiO_2_ and Kaolin to obtain 81 samples (including one sample of pure water). The component concentration is calculated as the ratio of the mass of component to that of the total mixture, and the component concentrations of Kaolin and TiO_2_ are denoted as *c*_k_ and *c*_t_ respectively. For calibration and prediction, the whole sample set is partitioned into two subsets. The training and prediction subsets comprise 72 (89%) and nine (11%) samples, respectively. The training subset is applied to build the LS-SVM model and the prediction subset is used to evaluate the model performance. In addition, a subset of additional samples, namely the inline test subset, is used to assess the performance of inline measurement. The distribution of samples is shown in Figure 3.

### 2.4. Ultrasonic Features

Both the amplitudes and shapes of ultrasonic signals change along with the variations of particle concentrations in multicomponent suspensions, so the amplitude and shape features are used as the potential features to predict the suspensions concentrations. All the ultrasonic features are summarized in Table 1. Time and frequency domain features are calculated from the time domain signals *x*(*n*) and the frequency spectrum X(*k*), respectively. *T*_1_–*T*_7_ and *T*_8_–*T*_12_ represent the amplitude and distribution of the time domain signals *x*(*n*), respectively. Similarly, *F*_1_–*F*_7_ and *F*_8_–*F*_12_ describe the amplitude and distribution of the frequency spectrum X(*k*) , respectively.

Furthermore, along with the increase of concentration, significant changes take place to the frequency amplitudes and the peak frequency at which the maximum frequency amplitude occurs [26]. So the peak frequency and some frequency amplitudes are also selected as features to detect the suspensions concentration. In addition, the ultrasonic velocity and attenuation coefficient of suspensions can be used to deduce the concentration [27]. The ultrasonic velocity *v*_s_ is defined as the acoustic path length (2*l*) divided by the time of flight of the ultrasonic signal *t*_s_. The attenuation coefficients of suspensions for time and frequency features are calibrated by measuring those of pure water and calculated by the following Equation (5):

(5) $$\alpha =\frac{1}{2l}\ln\left(\frac{A_{w}}{A_{s}}\right)$$

where *A*_w_ is one of the amplitudes of *T*_1_–*T*_7_ and *F*_1_–*F*_6_ in water, *A*_s_ is one of the amplitudes of *T*_1_–*T*_7_ and *F*_1_–*F*_6_ corresponding to *A*_w_ in suspensions.

**Table 1:** The features extracted from ultrasonic signals.

Features	Time Domain Features		Frequency Domain Features	
	Amplitude	Attenuation	Amplitude	Attenuation
Mean	$T_{1}=\frac{1}{N}\overset{N}{\underset{n=1}{\sum }}\left|x\left(n\right)\right|$	${\text{α}}_{T1}$	$F_{1}=\frac{1}{M}\overset{M}{\underset{k=1}{\sum }}\left|X\left(k\right)\right|$	${\text{α}}_{F1}$
Standard deviation	$T_{2}=\sqrt{\frac{1}{N}\overset{N}{\underset{n=1}{\sum }}{\left(x\left(n\right)-T_{1}\right)}^{2}}$	${\text{α}}_{T2}$	$F_{2}=\sqrt{\frac{1}{M}\overset{M}{\underset{k=1}{\sum }}{\left(X\left(k\right)-F_{1}\right)}^{2}}$	${\text{α}}_{F2}$
Root mean square	$T_{3}=\sqrt{\frac{1}{N}\overset{N}{\underset{n=1}{\sum }}{\left(x\left(n\right)\right)}^{2}}$	${\text{α}}_{T3}$	$F_{3}=\sqrt{\frac{1}{M}\overset{M}{\underset{k=1}{\sum }}{\left(X\left(k\right)\right)}^{2}}$	${\text{α}}_{F3}$
Square mean root	$T_{4}={\left(\frac{1}{N}\overset{N}{\underset{n=1}{\sum }}\sqrt{\left|x\left(n\right)\right|}\right)}^{2}$	${\text{α}}_{T4}$	$F_{4}={\left(\frac{1}{M}\overset{M}{\underset{k=1}{\sum }}\sqrt{\left|X\left(k\right)\right|}\right)}^{2}$	${\text{α}}_{F4}$
Energy	$T_{5}=\overset{N}{\underset{n=1}{\sum }}{\left(x\left(n\right)\right)}^{2}$	${\text{α}}_{T5}$	$F_{5}=\overset{M}{\underset{k=1}{\sum }}{\left(X\left(k\right)\right)}^{2}$	${\text{α}}_{F5}$
Extremum	$T_{6}=\text{max}\left(x\left(n\right)\right)$	${\text{α}}_{T6}$	$F_{6}=\text{max}\left(X\left(k\right)\right)$	${\text{α}}_{F6}$
	$T_{7}=\text{min}\left(x\left(n\right)\right)$	${\text{α}}_{T7}$	$F_{7}=f_{\text{peak}}$	
Latitude factor	$T_{8}=\frac{\text{max}\left(\left|x\left(n\right)\right|\right)}{T_{4}}$		$F_{8}=\frac{\text{max}\left(\left|X\left(k\right)\right|\right)}{F_{4}}$	
Crest factor	$T_{9}=\frac{\text{max}\left(\left|x\left(n\right)\right|\right)}{T_{3}}$		$F_{9}=\frac{\text{max}\left(\left|X\left(k\right)\right|\right)}{F_{3}}$	
Kurtosis	$T_{10}=\frac{\sum_{n=1}^{N}{\left(x\left(n\right)-T_{1}\right)}^{4}}{NT_{2}^{4}}$		$F_{10}=\frac{\sum_{k=1}^{M}{\left(X\left(k\right)-F_{1}\right)}^{4}}{NF_{2}^{4}}$	
Shape factor	$T_{11}=\frac{T_{3}}{T_{1}}$		$F_{11}=\frac{F_{3}}{F_{1}}$	
Impulse factor	$T_{12}=\frac{\text{max}\left(\left|x\left(n\right)\right|\right)}{T_{1}}$		$F_{12}=\frac{\text{max}\left(\left|X\left(k\right)\right|\right)}{F_{1}}$	
Other	$v_{\text{s}}=\frac{2l}{t_{s}}$		*X*(*f*) (*f*=1.1, 1.6, 1.9, 2.2, 2.5, 2.8, 3.1, 3.6 MHz)	${\text{α}}_{f}\left(\text{X}\left(f\right)\right)$

## 3. Results and Discussion

### 3.1. Data Pre-Processing

**Figure 4:**
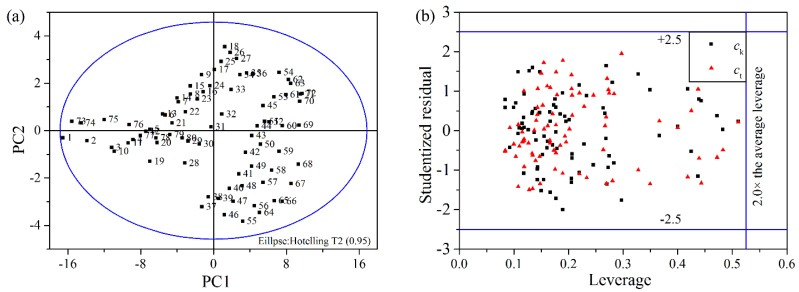
(**a**) The scatter plot of principal components (PC1/PC2); (**b**) The scatter plot of the leverage and the studentized residual.

Because of the potential influences of bubbles and impurities as well as the complex characteristics of suspensions in industrial process, the detection of outliers is very critical in data pre-processing [28]. In this paper, the scatter plot of the first two principal components (PC1 and PC2) is used to detect the potential outlier(s). The scatter plot is a map of the target values, displaying how the features are situated with respect to each other. The samples situated outside the tolerance ellipse based on Hotelling’s T2 are considered as outliers [29]. As shown in Figure 4a, no samples are flagged and demonstrated to be outliers and the samples are not overlapped and can be clearly separated in scatter plots.

Leverage analysis is also used as an additional diagnostic tool for outliers, which reflects differences between a sample spectrum and the average spectrum of the sample set [30]. The concentration residuals are scaled by the standard deviation of the residuals to generate the studentized residuals. The relationship between the leverage and the studentized residual is shown in Figure 4b. The outliers are flagged at ±2.5 studentized residuals and the double of the average leverage [31]. As shown in Figure 4b, no samples are flagged and demonstrated to be outliers in accordance with the analysis above.

There are 54 ultrasonic features for each sample. All features can be used to establish the LS-SVM model or the PLS model, but irrelevant or incorrect features may have adverse impacts on the generalization performance. The feature selection based on the importance of features in the model can not only reduce the dimensionality of data but also improve the prediction performance.

**Figure 5:**
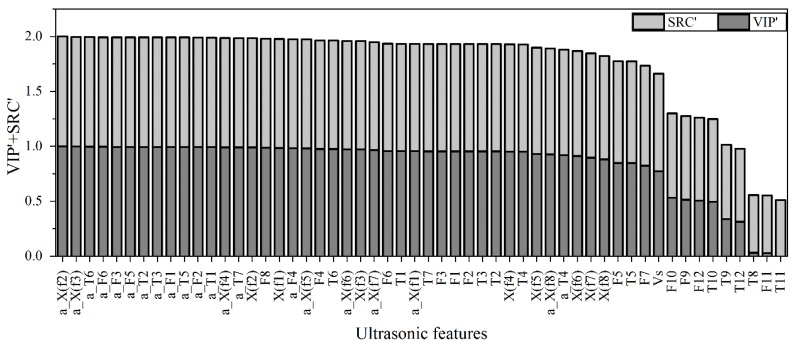
The importance of each feature.

The importance of features, which is calculated with a combination of VIP and SRC, serves as the basis of feature selection. The absolute values of VIP and SRC of each feature are normalized into the region [0, 1] and denoted as VIP’ and SRC’, respectively. The sum of VIP’ and SRC’ gives an indication of how important the features are to the target values. All features are arranged in the decreasing order of importance (Figure 5). It shows that time distribution features (*T*_8_–*T*_12_) and frequency distribution features (*F*_9_–*F*_12_) are insensitive or irregular in correlation to the particle concentrations.

According to the importance of the features, the unimportant features are excluded sequentially and new feature subsets (Table 2) are re-grouped. M1 subset contains all features (see Table 1). M2 and M3 subsets include all time domain and frequency domain features, respectively. M4 subset contains all time domain features excluding *T*_8_–*T*_12_, and M5 contains all frequency domain features excluding *F*_9_–*F*_12_. M6 subset is the combination of M4 and M5 subsets. All subsets are used to build the model and find out the optimal feature subset that shows the best performance.

**Table 2:** The feature subset summaries.

Subset	Time Features	Frequency Features	Count
M1	$T_{1}-T_{12},{\text{ α}}_{T1}-{\text{α}}_{T7},\;v_{\text{s}}$	$F_{1}-F_{12},{\text{ α}}_{F1}-{\text{α}}_{F6},\;X\left(f\right),{\text{ α}}_{f}\left(\text{X}\left(f\right)\right)$	54
M2	$T_{1}-T_{12},{\text{ α}}_{T1}-{\text{α}}_{T7},\;v_{\text{s}}$	/	20
M3	/	$F_{1}-F_{12},{\text{ α}}_{F1}-{\text{α}}_{F6},\;X\left(f\right),{\text{ α}}_{f}\left(\text{X}\left(f\right)\right)$	34
M4	$T_{1}-T_{7},{\text{ α}}_{T1}-{\text{α}}_{T7},\;v_{\text{s}}$	/	15
M5	/	$F_{1}-F_{8},{\text{ α}}_{F1}-{\text{α}}_{F6},\;X\left(f\right),{\text{ α}}_{f}\left(\text{X}\left(f\right)\right)$	30
M6	$T_{1}-T_{7},{\text{ α}}_{T1}-{\text{α}}_{T7},\;v_{\text{s}}$	$F_{1}-F_{8},{\text{ α}}_{F1}-{\text{α}}_{F6},\;X\left(f\right),{\text{ α}}_{f}\left(\text{X}\left(f\right)\right)$	45

### 3.2. Optimization of Model Parameters

According to the analysis above, the optimization of parameters γ and σ^2^ is the critical step to obtain the optimal LS-SVM model. In this study, the iterative grid search is performed to find the parameter range. For each combination of (λ, σ^2^) (grid point), the 10-fold cross-validation is used to calculate the prediction errors using the training set. The optimum parameters are selected which produce smallest prediction errors.

**Figure 6:**
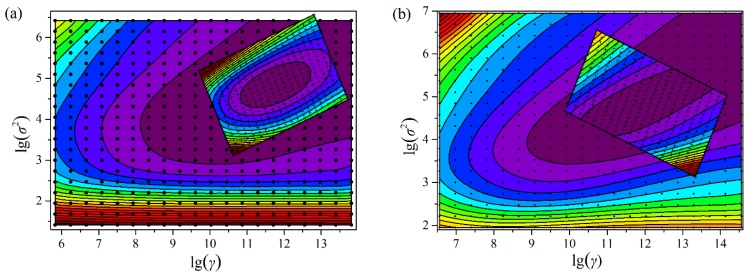
The contour plot of RMSEV *versus* γ and σ^2^ in the grid search for (**a**) *c*_k_ and (**b**) *c*_t_. The grids “·” and “+” are 10 × 10 in the first step and the second step, respectively. The color stand for the value of RMSEV.

The grid search is a gradual search process. The first step is a crude search with a large size, while the next step is conducted with a smaller size than the previous step until it meets the search conditions. The result of the grid search is an error surface spanned by the model parameters, and the color stand for RMSEV values. With the parameters in a smooth area, a robust model can be built. Hence, the optimal parameters are selected from a smooth subarea with the lowest RMSEV. After the grid search, the optimal combination of (λ, σ^2^) is achieved for the LS-SVM models. The contour plot for the optimization process of parameters γ and σ^2^ for M5 feature subset is shown in Figure 6. The search process has been completed through only two steps (two iterations). For the M5 feature subset, the optimal combinations of (λ, σ^2^) are found to be (1.1545 × 10^5^, 114.9) and (1.4978 × 10^5^, 125.5587) for *c*_k_ and *c*_t_, respectively. In the same way, the optimal combinations of (λ, σ^2^) for other feature subsets can be found.

**Figure 7:**
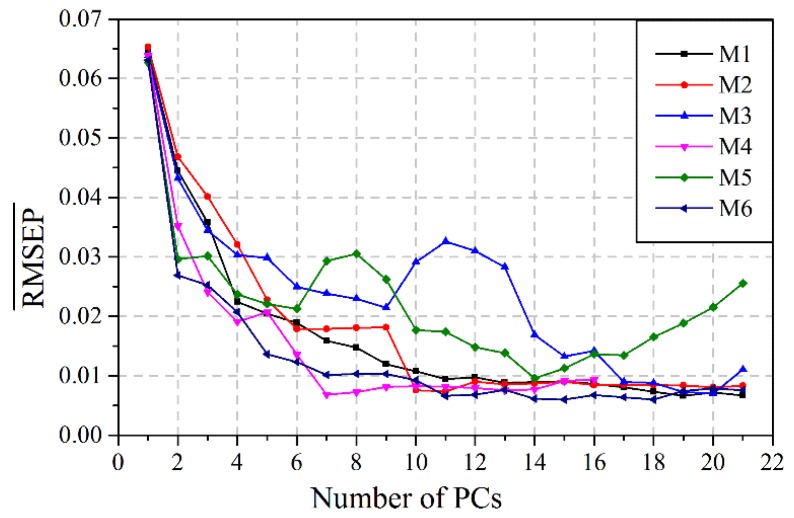
$\bar{\text{RMSEP}}$ of each feature subset against the number of PCs

The PLS model is used as the reference method in this study. The PLS model is a common and useful tool for modeling the relationship between the features and the target values. The model order (the number of PCs) of the PLS model is important to achieve good performance. $\bar{\text{RMSEP}}$, which is the average RMSEP of *c*_k_ and *c*_t_, is used to determine the optimal number of PCs. For each feature subset, the PLS model is built and $\bar{\text{RMSEP}}$ is calculated (Figure 7). It can be seen that all models start with high $\bar{\text{RMSEP}}$ values but decrease rapidly at first and then decrease smoothly. The $\bar{\text{RMSEP}}$ values become asymptotic around 10–15 factors, but the minimums are different. The optimum number of PCs which produces the smallest $\bar{\text{RMSEP}}$ can be easily found in Figure 7 and then used to construct the PLS model.

### 3.3. Model Training and Testing

Using the optimum parameters γ and σ^2^, the LS-SVM models for *c*_k_ and *c*_t_ are trained for each feature subset using the training subset, respectively. To investigate the prediction ability of the model, the model is used to measure the particle concentrations in prediction subset, which doesn’t contribute to the model building. The performance of the LS-SVM model ($R_{\text{P}}^{2}$ and RMSEP) is calculated and showed in Table 3. For the comparison among six feature subsets, the best performance is achieved with M5 subset. In the optimal model and selection of features, RMSEP of the LS-SVM model is 0.31 wt% for Kaolin and 0.34 wt% for TiO_2_. The maximum absolute residual errors $\text{max}\left(\left|y-\hat{y}\right|\right)$) in prediction subset of Kaolin and TiO_2_ are less than 0.45 wt% and 0.56 wt%, respectively. Based on the results above, it can be concluded that the combination of ultrasonic sensor and the LS-SVM model is a reliable and accurate method for the prediction of particle concentrations in multicomponent suspensions.

The PLS models are built using the same sample subsets as the LS-SVM models and the optimal parameters which have the smallest $\bar{\text{RMSEP}}$ shown in Figure 7. The performance comparison between the PLS model and the LSV-SVM model is shown in Table 3. RMSEP and R^2^ of the optimal PLS model, namely 0.52 wt% and 0.995 for *c*_k_, 0.67 wt% and 0.989 for *c*_t_, are larger and less than those of the LS-SVM model, respectively, which indicates that the prediction performance of the LS-SVM model is better than that of the PLS model. The PLS model is suited to the linear system, while the LS-SVM model is commonly used in the nonlinear system. Hence, the reason why the LS-SVM model produces higher prediction accuracy than the PLS model may be the presence of nonlinear relationship between ultrasonic features and component concentrations.

**Table 3:** The performance of the partial least square (PLS) model and the least squares support vector machines (LS-SVM) model.

Feature Subset	PLS				LS-SVM			
	RMSEP_*c*_k_	$R_{P}^{2}$_*c*_k_	RMSEP_*c*_t_	$R_{P}^{2}$_*c*_t_	RMSEP_*c*_k_	$R_{P}^{2}$_*c*_k_	RMSEP_*c*_t_	$R_{P}^{2}$_*c*_t_
M1	0.0068	0.993	0.0075	0.988	0.0057	0.994	0.0081	0.984
M2	0.0060	0.994	0.0098	0.977	0.0065	0.993	0.0091	0.980
M3	0.0058	0.996	0.0092	0.980	0.0056	0.994	0.0156	0.943
M4	0.0055	0.996	0.0089	0.982	0.0048	0.996	0.0088	0.982
M5	0.0069	0.993	0.0124	0.964	0.0031	0.999	0.0034	0.998
M6	0.0052	0.995	0.0067	0.989	0.0030	0.999	0.0041	0.996

In addition, through the selection of suitable model parameters, the LS-SVM model and the PLS model have good prediction capabilities. After excluding the non-informative and unimportant features, the prediction accuracies of both models are further improved. With all features (M1), the LS-SVM model performs slightly better than the PLS model. However, after feature selection (M4, M5 and M6 subsets), the LS-SVM model generates a better prediction performance than the PLS model. The PLS model and the LS-SVM model have the best prediction performances for M6 subset (44 features) and M5 subset (30 features), respectively. The further removal of features causes a reduction of the accuracy due to the lack of sufficient information for the model, and the further addition of features also degrades the performance because of the addition of redundant “noise” to the model. Hence, it can be concluded that the selection of appropriate features is crucial to the model performance, which can reduce the system dimension and improve the model accuracy.

**Figure 8:**
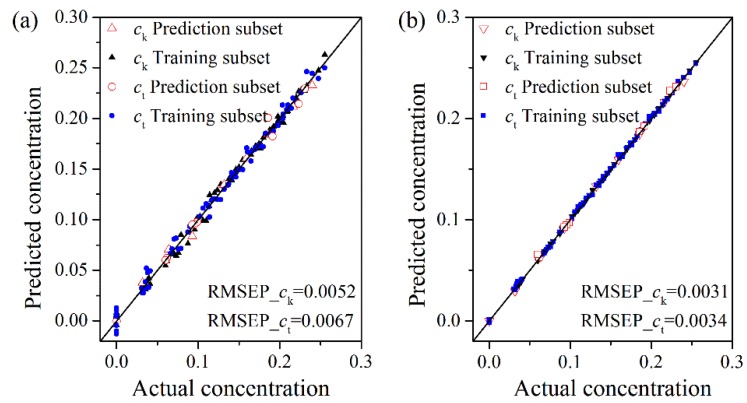
The predicted values *vs.* the actual values using (**a**) the optimal PLS model and (**b**) the optimal LS-SVM model.

With the optimal model parameters and optimal feature subset, the plots of actual concentrations against prediction concentrations for the training and prediction subsets using the PLS model and the LS-SVM model are shown in Figure 8. The results show the points tend to cluster along the lines close to the 45° tangent line, which reflects an acceptable model adequacy. It can be easily seen that the LS-SVM model gives both better fitting and prediction results. In the LS-SVM model, all points of the plots fall on or close to the unity line, which suggests that the prediction capability of the model is good and the over-fitting problem is not obvious. It can also be found that the prediction values of the PLS model have significant errors for the samples, which only contain one component in the suspensions. However, the LS-SVM model presents an excellent prediction capability in the full concentration range. The sudden changes of features occur when the components of suspensions change from two to three. The LS-SVM model is not only capable of modeling the main linear relationship but also can grasp the nonlinear relationship existing in real data, thus leading to more accurate predictions. The PLS model is inherently a linear modeling method, which makes it impossible to well account for the nonlinear relationship of the data.

### 3.4. Inline Measurement

The inline measurement of particle concentrations in multicomponent suspensions is of great importance. The combination of ultrasonic sensor and the LS-SVM model provides an alternative method to inline measure the particles concentration.

In order to evaluate the inline measurement ability of the model, an independent inline measurement is implemented. In this process, the real-time ultrasonic signals are acquired with an interval of 1 s after the suspensions become homogeneous. A time-moving window with a length of 10 s is used to average the signals. In the process, as the new data are added into the window, the oldest data are disposed. Then, the resulting signals are processed by the transformations mentioned above (average, window, zero-pad and FFT). The ultrasonic features are selected in accordance with the M5 subset. After auto scaling, the data set is statistically analyzed to determine outliers, which aim to decide whether the sample is within the scope of the model or not. With the optimal model parameters, the LS-SVM model is used to inline measure the intently-designed samples (the data are not used in model development, see Figure 3).

**Figure 9:**
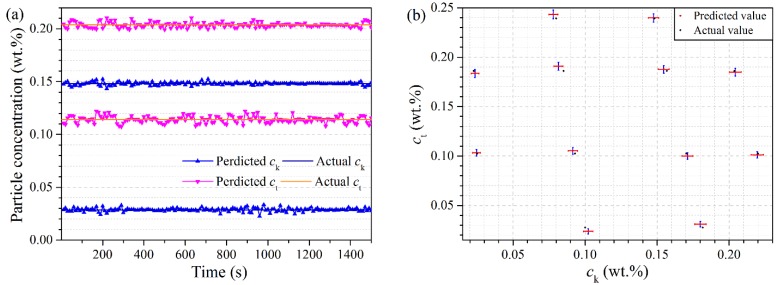
The result of inline measurement: (**a**) The real-time measurement concentration; (**b**) The XY error bar graph of the averaged *c*_k_ and *c*_t_ in 5 min.

The results of inline measurement are shown in Figure 9. As shown in Figure 9a, the concentrations of two real-time measurements are obviously fluctuant, and specifically, *c*_t_ shows more fluctuant changes than *c*_k_. Compared with the offline predicted results in Figure 8, the error of inline concentration measurement is larger, which is mainly caused by the dynamic measurement along with the fluctuation of signals. The noise disturbance cannot be eliminated through the mean method in consideration of real-time measurement, and accordingly the accuracy of concentration measurement is reduced. Nevertheless, the errors of concentration measurements of Kaolin and TiO_2_ in inline test subset are less than ±0.65 wt% and ±0.80 wt% respectively, which meet the industrial requirements.

In Figure 9b, the black points and the red points represent the actual concentrations and the averaged concentrations for 5 min, respectively. The error bars denote the concentration fluctuation in 5 min. The distance of two points in the same sample indicates the difference of two concentrations. As shown in Figure 9b, the small distance between two points in the same sample demonstrates that the measurement has high accuracy when the mean method is used. In the event that the low real-time requirements are put forward, the measurement accuracy can be further improved through increasing the collection points or slip averaging the measurement results. The inline measurement performance shows that the LS-SVM model is capable of modeling nonlinear system dynamically.

## 4. Conclusions

This paper proposes an alternative methodology for inline measurement of particle concentrations in multicomponent suspensions based on a combination of ultrasonic sensor and LS-SVM model. The optimum feature subset that results in the best performance is found based on SRC and VIP values. According to the result of model evaluation, the measurement system has an acceptable accuracy. In comparison with the PLS model, the LS-SVM model with fewer features has a better prediction ability of particle concentrations in multicomponent suspensions. Furthermore, inline measurement is performed and the results reveal that the measurement accuracy is sufficiently high in the application of monitoring industrial processes. The proposed technique can easily be extended to detecting the mixtures of more components and provides a tool for process control where the complex system makes it difficult to build a reliable physical model.

In the future, related research will be carried out to take the superposition phenomena caused by temperature into consideration, further improve the measurement accuracy by increasing samples, and develop the inline monitoring technology for production process and product quality based on the inline concentration measurement.
